# Age-specific associations with dental caries in HIV-infected, exposed but uninfected and HIV-unexposed uninfected children in Nigeria

**DOI:** 10.1186/s12903-022-02421-w

**Published:** 2022-09-27

**Authors:** Paul Akhigbe, Nneka M. Chukwumah, Morenike Oluwatoyin Folayan, Kimon Divaris, Ozoemene Obuekwe, Augustine Omoigberale, Elima Jedy-Agba, Michael Kim, Manhattan E. Charurat, Vincent P. Richards, Modupe O. Coker

**Affiliations:** 1 Institute of Human Virology Nigeria, Abuja, Nigeria; 2 Department of Preventive Dentistry, University of Benin Teaching Hospital, Benin, Edo State, Nigeria; 3 Department of Child Dental Health, Obafemi Awolowo University, Ile-Ife, Nigeria; 4 Division of Pediatric and Public Health, Adams School of Dentistry, University of North Carolina-Chapel Hill, Chapel Hill, NC USA; 5 Department of Epidemiology, Gillings School of Global Public Health, University of North Carolina-Chapel Hill, Chapel Hill, NC USA; 6 Department of Oral Biology, School of Dental Medicine, Rutgers University, Newark, NJ USA; 7 Institute of Human Virology, University of Maryland School of Medicine, Baltimore, MA USA; 8 Department of Biological Sciences, Clemson University, Clemson, SC USA; 9 Department of Epidemiology, School of Public Health, Rutgers University, Newark, NJ USA; 10 Department of Epidemiology, Geisel School of Medicine at Dartmouth, Hanover, NH USA; 11 Child Health Department, University of Benin Teaching Hospital, Benin, Edo State Nigeria; 12 Department of Oral and Maxillofacial Surgery, University of Benin Teaching Hospital, Benin, Edo State Nigeria

**Keywords:** HIV, Caries, Dental health, Children, Hypoplasia, CD4

## Abstract

**Background:**

HIV infection and its management confer a substantial health burden to affected individuals and have been associated with increased risk of oral and dental diseases. In this study, we sought to quantify HIV-associated differences in the prevalence and severity of dental caries in the primary and permanent dentition of 4–11-year-old Nigerian Children.

**Methods:**

We used clinical, laboratory, demographic, and behavioral data obtained from an ongoing cohort study of age-matched HIV-infected (HI, n = 181), HIV-exposed-but-uninfected (HEU, n = 177), and HIV-unexposed-and-uninfected (HUU, n = 186) children. Measures of dental caries experience (i.e., prevalence and severity) were based on dmft/DMFT indices recorded by trained and calibrated clinical examiners. Differences in primary and permanent dentition caries experience between HI, HEU, and HUU were estimated using multivariable logistic and negative binomial regression modeling.

**Results:**

HI children had significantly higher caries experience (33%) compared to HEU (15%) and HUU (22%) children. This difference persisted in fully adjusted analyses [odds ratio (OR) = 1.6; 95% confidence interval (CI) = 1.0–2.6], was most pronounced in the permanent dentition (OR = 3.4; 95% CI = 1.2–9.5), and mirrored differences in caries severity. While molars were predominantly affected in both primary and permanent dentitions, caries lesion patterns differed between dentitions. Caries severity was significantly associated with hypoplastic primary teeth, gingival inflammation, and lower CD4 counts.

**Conclusions:**

We found that the higher prevalence and severity of dental caries among HI children was driven by increased burden of permanent dentition caries compared to their uninfected counterparts. The dentition-specific associations identified in this study highlight the need to design and implement age-specific caries prevention strategies. These may include intensified oral hygiene regimens aimed at mitigating the cariogenic impact of hyposalivation among HI children. Similarly, the long-lasting impacts of developmental defects of the enamel in the primary and permanent dentitions must not be ignored.

**Supplementary Information:**

The online version contains supplementary material available at 10.1186/s12903-022-02421-w.

## Introduction

The oral health of children living with HIV has been the focus of several recent investigations [1–6]. Oral pathologies including candidiasis, necrotizing ulcerative gingivitis, necrotizing ulcerative periodontitis, necrotizing stomatitis, hairy leukoplakia, and oral ulcers have been well characterized among HIV-infected (HI) individuals. However, childhood caries, oral mucosal lesions, and periodontal disease have been studied less [7, 8]. This represents a knowledge gap, because dental caries is the most prevalent disease globally, and the most common oral disease in children [1, 9]. Early childhood caries (ECC) is the tenth most prevalent disease globally; and when untreated, can be painful, expensive to treat, and can lead to tooth loss, impaired nutrition [10, 11] and a diminished quality of life [1, 9, 12].

We and other researcher teams have reported observations of higher levels of ECC in HI compared to uninfected children [4, 13–18]. These higher rates for caries may be due to the higher rates of hyposalivation and hypoplasia observed in PLWH [14] when compared to their uninfected counterparts; compounded by the sustained consumption of sucrose-based (antiretroviral therapy) ART syrups and suspensions, hypoplastic teeth due to the immunological impact of HIV infection [17, 18], low salivary flow [19] and cariogenic oral microbiota, including bacterial-fungal components of dysbiosis [20–23]. In spite of this emerging evidence, there is no clear consensus on whether the impact of perinatal HIV infection on caries risk in the primary dentition persists, diminishes, or accentuates in the permanent dentition.

Recent studies compared HI children to uninfected children [3, 4, 13, 18, 24, 25] while only one study included a comparison with HIV-exposed-but-uninfected (HEU) children [9]. Our earlier findings suggested that the immune status of HI (as reflected in CD4 + percentages and counts) had the greatest influence on the differences in salivary community composition between HI and uninfected children [20]. These initial observations were based on studies including the primary dentition in early childhood. In the present study, we sought to expand upon earlier findings, and examine the relationship between caries and HIV infection in older children and including the mixed dentition. Accordingly, we sought to quantify HIV infection-associated differences in the prevalence and severity of dental caries in the primary and permanent dentition in a cohort of 544; 4–11-year-old Nigerian children who were HI, HIV-exposed-but-uninfected (HEU), and HIV-unexposed-and-uninfected (HUU). Additionally, we studied the influence of age-specific factors such as dentition and duration on ART on the association between HIV exposure/infection and caries. Our hypothesis was that compared to HUU children, caries experience of HI children would be higher, and this association would persist in both primary and permanent teeth.

## Methods

Dental Caries and its association with Oral Microbiomes and HIV in young children—Nigeria (DOMHaIN) study is a prospective cohort study of young children in Nigeria aimed at investigating aspects of the dental caries-associated microbiome in children infected or exposed to HIV [26]. Eligible children were recruited from the University of Benin Teaching Hospital (UBTH), Edo State, Nigeria, in three groups: HI, HEU, and children that were unexposed and uninfected (HUU). HI, children receiving treatment at the Special Treatment Clinic (STC) within the eligible age range were approached for recruitment. Children who were uninfected but receiving care at the STC were recruited as HEU children (i.e., perinatally exposed to HIV) after confirmation of HIV status of the mother at the time of birth. Additional HI and HEU children were recruited from the HIV/AIDS pediatric clinic or by referral by mothers attending adult ART clinic at UBTH. Age-matched HUU children were recruited from well-child and pediatric clinics at UBTH.

### Inclusion and exclusion criteria

Children aged 4 to 11 years, whose parents provided permission and informed consent were invited to participate in the study. Children’s assent was sought from children 8 years and older. Children with physical, clinical, or mental incapacity were excluded.

### Sample size

With a prevalence of dental caries of 30% [4], statistical significance and power of 0.05 and 0.80 respectively, and a 10% surplus to accommodate for invalid responses, a minimum sample of 276 participants (138 in each group) was required for a least detectable difference of 15% between HI and HUU children.

### Study procedures

Identification and enrollment of study participants took place over an eight-month period from May through December 2019. Medical records, questionnaires, and oral examinations were performed at baseline, at six months and 1-year post enrollment. Results from the study’s baseline data are only presented here. Institutional review boards at UBTH, University of Maryland Baltimore (HP-00084081) and Rutgers State University of New Jersey (Pro2019002047), gave approval for this study.

Caries experience was the study’s analytical endpoint, with dependent variables being caries prevalence (i.e., binary) and dmft/DMFT (i.e., continuous) indices. The three study groups (i.e., group membership is the independent variable) were defined by HIV infection and exposure. All other demographic, behavioral, and clinical characteristics variables were treated as confounders. Demographic information (i.e., sex at birth and age in years) and social characteristics (i.e., maternal age, education, and employment status) were self-reported via questionnaires completed by parents; in cases where parents were deceased or otherwise unavailable, information was retrieved from caregivers or clinical chart review. Information on oral health history (i.e., previous dental treatments), oral hygiene practices (i.e., tooth brushing frequency), and medication history (i.e., ART or antibiotic exposure) was collected via interviews. With respect to medical history, interview data were confirmed or resolved by chart review. Anthropometric measurements and medication use were also documented in medical records. All questionnaires and data reviews were conducted by trained and certified staff according to standardized protocols.

#### Ascertainment of HIV infection

To accurately identify groups, HIV infection or exposure was determined via a review of maternal and child medical records, as well as a HIV confirmatory test of the child-participant at time of enrollment based on the National Protocols for HIV Testing Services (National Agency for the Control of AIDS) [27]. HIV infection status was determined from blood samples collected using the COBAS^®^ AmpliPrep/COBAS^®^ TaqMan^®^ HIV-1 Qualitative Test, version 2.0 (TaqMan^®^ HIV-1 Qual Test version 2.0), a dual-target total nucleic acid real-time PCR assay. HI status was also confirmed at the STC by participants’ attending physicians and confirmation with a rapid antibody test.

### Study measures

Medical and dental history, demographic data, baseline dietary information and oral health/caries assessment were obtained with the aid of a well-structured questionnaire at all visits. Caregivers were interviewed using standardized questionnaires for sociodemographic characteristics of the child, feeding, and oral hygiene practices. Maternal and infant medical records, questionnaires, and oral examinations provided were used in tandem with R to analyze the different factors that contributed to the prevalence of caries in HI, HEU, and HUU children in Nigeria. Medical history was obtained from these interviews and confirmed or resolved by chart review. Birth weight, current weight, height, and medication use were documented from medical records while maternal education and employment status were also assessed. Demographic information and personal characteristics were assessed via self-report questionnaires. Questionnaires collected information on demographic factors, medical and oral health history, oral hygiene practices, dietary intake (via Food Frequency Questionnaire), and medications. Salivary flow rate assessments (to ascertain hyposalivation) were implemented as previously published [26]. Briefly, participants were asked to lie on the dental chair with their head raised up. A wooden spatula was used to suspend the upper and the lower teeth and whole unstimulated saliva was allowed to form a pool in the mouth for five minutes. It was thereafter aspirated with the aid of sterile plastic pasteur pipette into a graduated 15 ml Falcon tube, and the volume was measured and recorded. The recorded value was divided by the time required for saliva production (5 mins) and recorded as the salivary flow rate. Hyposalivation was based on salivary flow rate lower than 0.2 ml/min.

#### Oral examination

Consenting participants were invited to a dental office for oral examination. The number of primary and permanent teeth present were recorded. Oral examination included assessments of gingival inflammation using the Gingival Index (GI) of Löe and Silness [28] based on index teeth; 11, 16, 26, 31, 36, 46 for permanent teeth and 51, 55, 65, 71, 75, 85 for primary teeth. GI scores were categorized as good (0.0–1.2), fair (1.3–3.0) and poor (> 3.0) oral hygiene. All teeth were also examined for stains, discolorations, fractures, and development defects of the enamel.

Enamel defects were identified, after plaque removal, using the modified DDE Index recommended by the World Dental Federation [29]. The index includes specific types of defects including demarcated and diffuse opacities, hypoplastic defects, and combinations. For this study’s purposes, we considered all defects ensemble as *enamel defects*. No radiographs were obtained or used in this study and tooth nomenclature followed the Fédération Dentaire Internationale (FDI) system.

A comprehensive caries assessment was performed for participating children by three dentists using the National Institute of Dental and Craniofacial Research (NIDCR) criteria [30]. Prior to commencement of the study, the Paediatric dentist (NMC) had interactive sessions with the examiners using pictures to assess the ICDAS staging of dental caries noting the presence of white spot lesions to frank cavitation in enamel and those extending to dentine. Inter-examiner and intra-examiner reliability assessments were then carried out among the trained examiners on six children with various stages of dental caries (ICDAS caries severity scores) on three different occasions one week apart. The intra-examiner Cohen Kappa scores for each examiner was 0.96, 0.92 and 0.84 respectively. The inter-examiner Cohen’s *kappa* value between the three examiners was excellent (0.91) using the pediatric dentist as the gold standard.

Caries experience was measured via standardized oral clinical examinations that were conducted blindly (i.e., without the examiners knowing who had HIV infection, who was exposed to HIV, or neither). During the examination, all teeth present in the oral cavity were assessed with the aid of artificial light, compressed air to dry the tooth surface, a dental mirror and a blunt dental probe to detect cavitated lesions. Caries detection was based on modified ICDAS criteria and children were further classified as either “caries free” (scores 0 and 1) or “caries affected” (based on the presence of at least one carious lesion on any tooth surface in the mouth with ICDAS > 1 (i.e., excluding “white spot lesions”), or surfaces restored or extracted due to caries. Similarly, quantitative caries presence at the person-level was defined as the sum of cavitated caries lesions (excluding early-stage, “white spot” lesions), missing (due to caries), or restored (“filled”) tooth surfaces primary or permanent teeth, for the dmft and the DMFT index, respectively.

#### Blood measures

Five ml of whole blood were obtained from each participant for CD4 + and CD8 + T cell count measurement via flow cytometry. Viral load determination was performed for HI children using real time polymerase chain reaction (RT-PCR).

### Measurement of covariates

Information regarding each child’s sociodemographic profile (age and sex), clinical status, birth factors (gestational age, delivery mode) and oral hygiene practices were collected via medical chart reviews and structured questionnaires. Maternal age, education and employment status were also collected and recorded from mother or caregiver (in cases where mother was dead or unavailable).

### Analytical approach

We estimated the association of demographic and clinical characteristics such as age, sex, delivery mode, gestational age, type of dentition, enamel defects, and salivary flow with caries experience. Tooth-specific data were then categorized into either primary or permanent teeth. Caries experience was classified as enamel or dentin caries in the initial oral examination. Bar plots were created to illustrate the distribution of caries experience.

To determine differences between the three HIV groups, associations between categorical variables were assessed using Pearson’s Chi-square or Fisher’s exact test where appropriate. For continuous variables, a *t*-test or ANOVA was performed. The prevalence (relative proportion with caries) and severity of caries experience among the three groups were estimated using the dmft/DMFT indices. Unadjusted odds ratios (OR) for the associations between caries and HIV infection/exposure were calculated. Adjusted OR for caries experience were obtained from logistic regression models controlling for important confounders and allowing for effect measure modification by age. Due to the skewed distribution of the dmft/DMFT indices, we used negative binomial regression models to estimate the association between perinatal HIV infection or exposure on caries severity. All analyses were performed using R and STATA^®^.

## Results

The study population comprised 544 unrelated children with mean age of 7 years. Half of participating children were male, 181 (33%) were HI, 177 (33%) HEU, and 186 (34%) were HUU children (Table 1). Sixty-eight mothers were deceased at the time of enrollment. Among those for which we had records or who are still living, most (85%) had at least a primary school education and were married. Children in the 3 study groups differed significantly with respect to mode of delivery, early infant feeding, birth weight, current anthropometric measurements and CD4 counts. There were no significant associations of HIV infection and exposure status with sex, socioeconomic status or age (Table 1).

**Table 1:** Socio-demographic, behavioral and clinical characteristics by Study Group

	All (N = 544)	HI (N = 181)	HEU (N = 177)	HUU (N = 186)	*P* value
*Child demographics*					
Age in months, mean (range)	86 (41–127)	87 (41–126)	86 (42–126)	86 (42–127)	0.75
Male, n(%)	290 (53)	87 (48)	95 (54)	108 (58)	0.16
*Maternal/early infant characteristics*					
Maternal age at delivery, mean (SD)	31.5 (7)	31.3 (5.8)	32.1 (6.6)	31.0 (7.2)	0.3
*Maternal educational level, n(%)*					**< 0.0001**
None/never been to school	82 (15.1)	70 (38.7)	5 (2.8)	7 (3.8)	
Primary school	92 (16.9)	38 (21.0)	42 (23.7)	12(6.5)	
Secondary school	202 (37.1)	57 (31.5)	83 (46.9)	62 (33.3)	
Above secondary	149 (27.4)	8 (4.4)	43 (24.3)	98 (52.7)	
Missing	19 (3.5)	8 (4.4)	4 (2.3)	7 (3.8)	
*Maternal employment status, n(%)*					**< 0.0001**
Professional	137 (25.2)	64 (35.4)	25 (14.1)	48 (25.8)	
Clerical	44 (8.1)	2 (1.1)	14 (7.9)	28 (15.1)	
Skilled manual	142 (26.1)	31 (17.7)	55 (31.1)	56 (29.6)	
Unskilled work	172 (31.6)	64 (35.4)	69 (39.0)	39 (21.0)	
Unemployed	13 (2.4)	6 (3.3)	7 (4.0)	0 (0)	
Others	18 (3.3)	7(3.9)	3 (2.3)	7 (3.8)	
*Maternal marital status, n(%)*					0.10
Married	463 (85.1)	147 (84.7)	148 (86.6)	168 (93.9)	
Separated or divorced	30 (5.5)	13 (7.4)	13 (7.0)	4 (2.2)	
Single never married	5 (0.9)	3 (1.7)	2 (0.6)	0 (0)	
Widow	27 (5.0)	11 (6.3)	10 (5.2)	6 (3.3)	
Others	1 (0.2)	0 (0)	0 (0.6)	1 (0.6)	
*Mother’s duration on ART in years, mean (SD)*	79.8 (60.0)	50.6 (47.3)	98.6 (59.9)	–	**< 0.0001**
*Vaginal delivery, n(%)*	461 (85)	166 (92)	144 (81)	151 (81)	**0.01**
*Gestational age, mean (SD)*	38.5 (3.3)	38.1 (4.8)	38.7 (2.1)	38.6 (2.2)	0.22
*Birth weight, mean (SD)*	3.10 (0.6)	3.04 (0.6)	3.06 (0.6)	3.18 (0.6)	**0.05**
*Exclusively breast-fed, n(%)*	255 (47)	63 (35)	78 (44)	114 (61)	**< 0.0001**
*Duration of breastfeeding in months, mean (SD)*	12 (6)	12 (5)	8 (7)	14 (5)	**< 0.0001**
*Deceased mother, n(%)*	68 (12.5)	62 (34.3)	3 (1.70)	3 (1.60)	**< 0.0001**
*Anthropometric characteristics*					
Weight in kg, mean (SD)	23.1 (6.8)	21.5 (5.5)	23.1 (7.4)	24.7 (7.1)	**< 0.0001**
Height in cm, mean (SD)	120 (14.1)	117 (13.6)	120 (14.4)	123 (13.8)	**< 0.0001**
Weight-for-age z score, mean (SD)	– 0.29 (1.2)	– 0.77 (1.2)	– 0.30 (1.2)	0.16 (1.2)	**< 0.0001**
Height-for-age z score, mean (SD)	– 0.33 (1.6)	– 0.95 (1.5)	– 0.24 (1.6)	0.19 (1.6)	**< 0.0001**
BMI Z score, mean (SD)	– 0.15 (1.7)	– 0.32 (1.4)	– 0.22 (1.8)	0.10 (1.9)	**< 0.0001**
*Current CD4 count in mm*^*3*^, *mean (SD)*	967 (486)	828 (520)	1010 (381)	1070 (512)	**< 0.0001**
* Frequency of brushing, n(%)*					**0.04**
Once a day	446 (82.0)	153(84.5)	155 (87.6)	138 (74.2)	
More than once a day	93 (17.1)	26(14.4)	22 (12.4)	45 (24.2)	
*Visited a dentist 12 months prior, n(%)*	14 (2.6)	4 (2.2)	2 (1.1)	8 (4.3)	0.15
*HIV infected children only*					
Duration on ART in months, mean (SD)		46.4 (31.6)			
Current viral load, mean (SD, [range])		20,675 (71,932, [0,647,780])			
Detectable viral load, n(%)		109 (60.2)			
*ART regimen type*					
AZT/3TC/EFV		145 (80)			
ABC/3TC/EFV		4 (2.2)			
ABC or TDF/3TC/DTG		17 (9.4)			
LPV/r-based regimen		13 (7.2)			

*P* values annotated in bold reflect significance i.e., < 0.05

None of the participating children had undergone dental extractions or received dental treatment prior to our study; meanwhile, 129 (24%) had at least one caries lesion and a third of those had decay visibly involving the dentine. Additional file 1: Table S1 highlights the additional dental findings across study groups. Among children > 6 years of age, 100/376 were diagnosed with caries compared to 29/168 among those aged ≤ 6 years (27% vs. 17%, *p* = 0.024). Among caries-affected children aged > 6, 85% had at least one primary carious tooth (i.e., overall, 23% caries prevalence in primary teeth among children aged > 6 years) while, as expected, all caries-affected children aged 6 years or younger had at least one primary carious tooth. In terms of quantitative caries experience, study participants had an average of 5 (SD = 8.4) caries-affected teeth, including 2.6 in the primary dentition and 2.9 in the permanent dentition. Among caries-affected children, the dmft index ranged from 1 to 10, while the DMFT index ranged from 1 to 8.

The prevalence of caries was highest in HI compared to HEU (*p* = 0.03) and HUU (*p* = 0.02) children. Notably, HEU children had a lower prevalence than HI or HUU children (12% vs. 22%, *p* = 0.03, Additional file 1: Table S1). The prevalence of caries lesions involving dentine was significantly higher in HI children compared to their HEU counterparts (24% vs 8%; *p* < 0.0001) and HUU counterparts (24% vs 15%; *p* < 0.0001). While these patterns remained consistent when examining age-specific groups, the strongest difference in caries experience between HIV groups was observed in children aged > 6 years (*p* = 0.002).

In further investigating these age-specific associations, we sought to evaluate dentition-specific differences. Table 2 highlights the independent association between study groups and caries prevalence in the primary and permanent dentition based on results from simple and multivariable logistic regression models. HIV infection remained independently associated with caries in the permanent dentition (*p* = 0.005). There was no significant association between HIV infection and sex; however, the association between age and hyposalivation differed by type of dentition. Enamel defects was an independent significant risk indicator for caries prevalence in the primary dentition (Table 2).

**Table 2:** Odds of dental caries by HIV exposure or infection status: Results from unadjusted and adjusted logistic regression models

	Caries in any dentition		Caries in primary dentition		Caries in permanent dentition	
	Unadjusted OR (95% CI)	Adjusted OR (95% CI)	Unadjusted OR (95% CI)	Adjusted OR (95% CI)	Unadjusted OR (95% CI)	Adjusted OR (95% CI)
Study group						
HI	**1.65 (1.04–2.61)**	**1.58 (0.96–2.59)**	1.42 (0.89–2.29)	1.41 (0.85–2.35)	**3.94 (1.55–10.00)**	**3.44 (1.25–9.49)**
HEU	**0.57 (0.33–0.98)**	**0.53 (0.30–0.92)**	**0.50 (0.29–0.88)**	**0.47 (0.26–0.85)**	1.42 (0.48–4.18)	1.29 (0.42–3.98)
HUU	Ref	Ref	Ref	Ref	Ref	Ref
Sex						
Female	0.99 (0.67–1.47)	0.99 (0.64–1.52)	0.97 (0.64–1.46)	0.96 (0.61–1.49)	0.75 (0.37–1.50)	0.73 (0.32 –1.64)
Male	Ref	Ref	Ref	Ref	Ref	Ref
Age (in months)	**1.01 (1.00–1.02)**	**1.01 (1.00–1.02)**	**1.01 (1.00–1.01)**	1.00 (0.99–1.01)	**1.04 (1.02–1.06)**	**1.04 (1.02–1.06)**
Gingival index	**3.11 (1.90–5.07)**	**2.72 (1.62–4.59)**	**2.43 (1.48–4.00)**	**2.21 (1.30–3.76)**	**4.62 (2.40–8.92)**	**3.10 (1.49–6.45)**
CD4 count						
< = 500 cells/mm^3^	1.52 (0.87–2.66)	0.82 (0.43–1.55)	1.28 (0.71–2.32)	0.75 (0.39–1.47)	**2.22 (0.96–5.10)**	0.76 (0.28–2.07)
> 500 cells/mm^3^	Ref	Ref	Ref	Ref	Ref	Ref
Enamel defects	**3.72 (2.03–6.82)**	**3.07 (1.62–5.83)**	**3.67 (1.99–6.75)**	**3.18 (1.66–6.09)**	**4.17 (1.83–9.52)**	**2.82 (1.04–7.62)**
Xerostomia	1.87 (0.54–6.48)	–	1.41 (0.37–5.40)	0.63 (0.13–2.93)	**5.87 (1.49–23.20)**	2.99 (0.54–16.43)
No prior visit to a dentist	0.40 (0.14–1.16)	–	**1.01 (1.00–1.03)**	0.68 (0.15–3.03)	2.56 (0.55–11.94)	1.00 (0.97–1.03)
Breastfeeding duration						
> 6 months	1.41 (0.86–2.30)	–	1.52 (0.91–2.57)	–	1.27 (0.54–2.99)	–
< = 6 months	Ref		Ref		Ref	Ref

*p values* < *0.1 are in bold fonts*

**Fig. 1:**
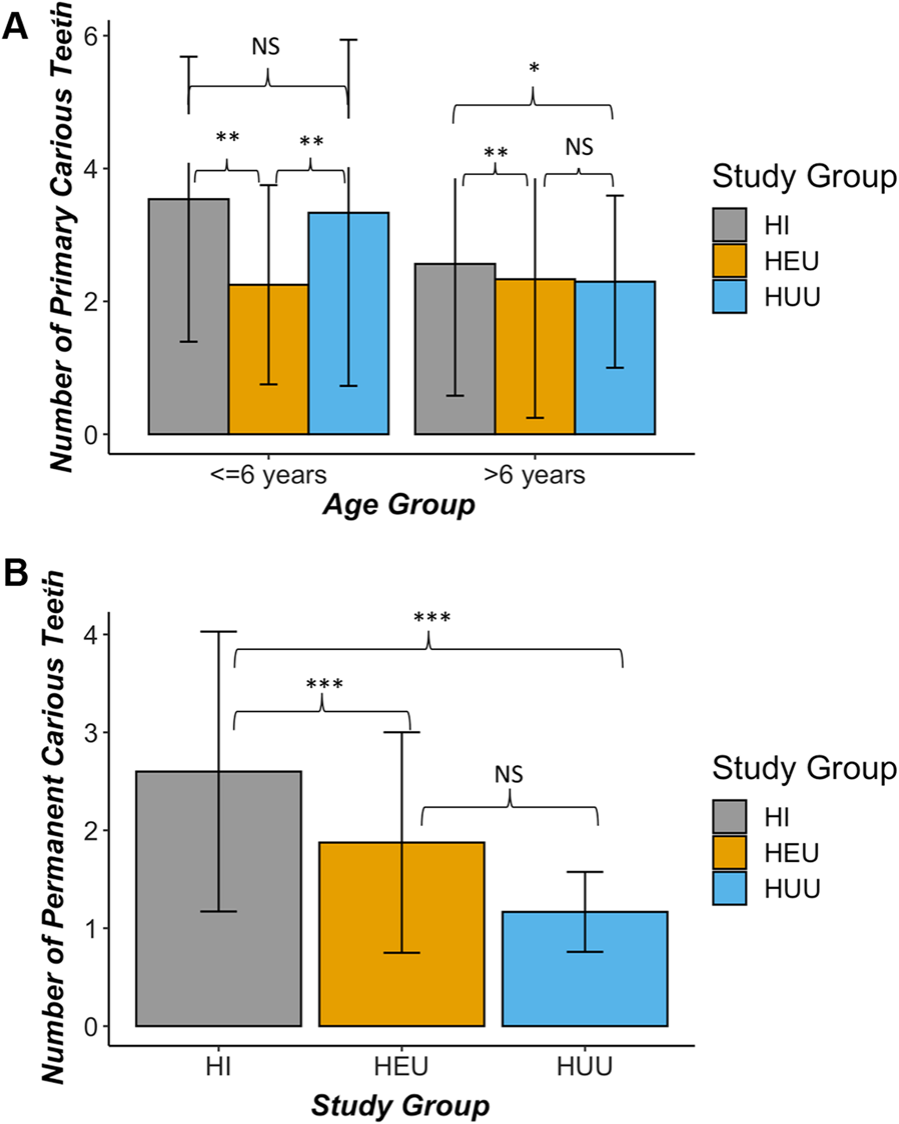
Caries severity varied by dentition, age and study group. **A** Caries severity measured as number of decayed teeth in **A** primary dentition by age and study group (N = 115); **B** Caries severity in permanent dentition by study group (N = 35)

Caries severity differed by type of dentition and study group (Fig. 1A, B). Compared to HUU, HEU children had a significantly lower dmft indices in the primary dentition (beta coefficient = − 0.29, *p* = 0.04); Table 3). Age was not associated with the number of carious teeth in primary dentition. In permanent teeth, caries severity was associated with HIV infection [adjusted mean ratio, AMR (95% confidence interval (CI); 5.4 (1.4–20.3)], hyposalivation and age. The presence of enamel defects was consistently associated with caries severity in both dentitions.

**Table 3:** Association between caries burden and HIV exposure/infection: Results from negative binomial models

	# of carious teeth in any dentition		# of carious teeth in primary dentition		# of carious teeth in permanent dentition	
	Unadjusted mean ratio (95% CI)	Adjusted mean ratio (95% CI)	Unadjusted mean ratio (95% CI)	Adjusted mean ratio (95% CI)	Unadjusted mean ratio (95% CI)	Adjusted mean ratio (95% CI)
Study group						
HI	**1.89 (1.13–3.17)**	**1.74 (0.92–3.31)**	1.43 (0.83–2.47)	1.35 (0.69–2.65)	**8.81 (2.90–26.76)**	**5.39 (1.43–20.28)**
HEU	**0.62 (0.36–1.08)**	**0.55 (0.32–0.96)**	**0.51 (0.28–0.92)**	**0.47 (0.26–0.85)**	2.25 (0.68–7.46)	2.28 (0.67–7.80)
HUU	Ref	Ref	Ref	Ref	Ref	Ref
Sex						
Female	0.95 (0.60–1.48)	0.94 (0.61–1.44)	0.97 (0.60–1.56)	0.92 (0.58–1.46)	0.85 (0.35–2.09)	0.81 (0.34–1.97))
Male	Ref	Ref	Ref	Ref	Ref	Ref
Age (in months)	1.00 (0.99–1.01)	1.00 (0.99–1.01)	1.00 (0.99–1.01)	1.00 (0.99–1.01)	**1.06 (1.03–1.09)**	**1.05 (1.02–1.08)**
CD4 count						
< = 500 cells/mm^3^	**1.76 (1.02–3.05)**	0.97 (0.49–1.94)	1.56 (0.87–2.78)	1.01 (0.49–2.10)	**2.67 (0.94–7.62)**	0.69 (0.20–2.35)
> 500 cells/mm^3^	Ref	Ref	Ref	Ref	Ref	Ref
Gingival inflammation						
Yes	**1.54 (0.99–2.41)**	**1.76 (1.12–2.75)**	**1.60 (1.00–2.56)**	**1.73 (1.08–2.78)**	1.36 (0.55–3.33)	1.72 (0.69–4.28)
No						
Hypoplastic teeth	**3.35 (1.64–6.84)**	**2.85 (1.39–5.81)**	**3.35 (1.59–7.06)**	**3.06 (1.40–6.70)**	**3.33 (0.81–13.74)**	**3.83 (1.03–14.20)**
Xerostomia	2.52 (0.57–11.21)	0.72 (0.16–3.27)	1.49 (0.29–7.51)	0.42 (0.08–2.21)	**6.73 (0.43–105.73)**	1.84 (0.12–28.83)
No prior visit to a dentist	2.83 (0.76–10.45)	2.31 (0.60–8.93)	2.31 (0.58–9.26)	–	4.81 (0.40–57.83)	–
Breastfeeding duration						
> 6 months	0.93 (0.55–1.57)	–	1.08 (0.64–1.82)	–	0.96 (0.34–2.78)	–
< = 6 months	Ref		Ref		Ref	

*p* values < 0.1 are in bold fonts

**Fig. 2:**
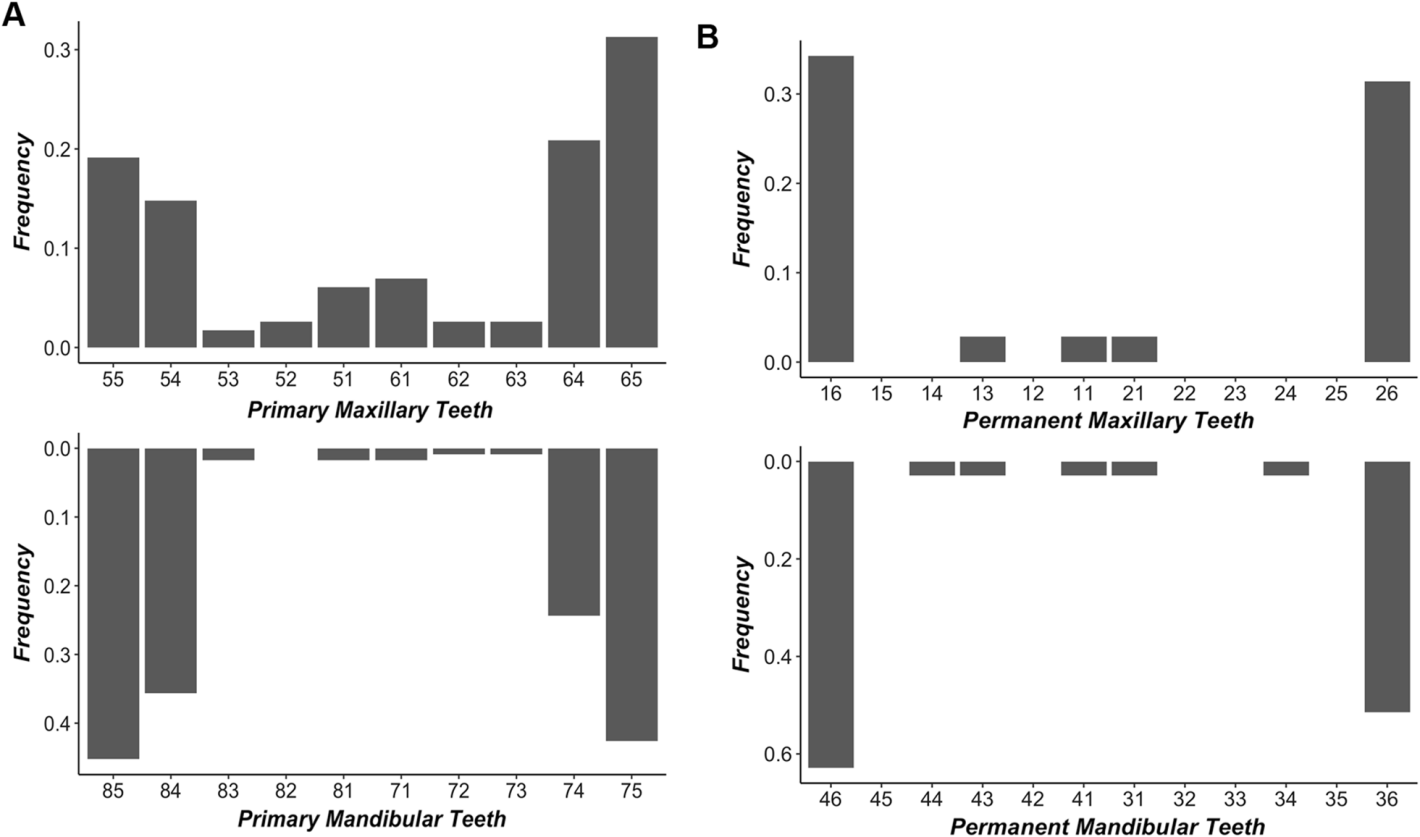
Disease frequency for specific teeth is distinct for primary and permanent dentition. **A** Distribution of specific carious primary maxillary and mandibular teeth. **B** Distribution of specific carious permanent maxillary and mandibular teeth

Greater caries severity scores of the permanent dentition were observed in HI compared with HUU with predominance in molars (Fig. 2A, B). Caries prevalence was significantly greater among participants with low (≤ 500 cells/mm^3^) compared to higher CD4 counts (OR = 2.2, 95% CI, 1.0–5.1). However, in all multivariable analyses where study groups were excluded (due to collinearity with immune status), CD4 + T cell measurements were independently associated with caries prevalence or severity (Additional file 1: Tables S2 and S3).

## Discussion

To our knowledge, this is the first comparative study of HI, HEU and HUU children investigating the association of perinatal HIV exposure and infection with dental caries in school-aged children or in children with mixed dentition. Our study results showed that the positive association of perinatal HIV infection with dental caries was more pronounced in children with permanent dentition (> 6 years of age). A more extensive analysis revealed that not only did HI children have significantly greater caries prevalence, but they also had a higher caries experience in terms of the number of caries-affected teeth. We noted distinct patterns of caries experience in the primary and the permanent dentitions highlighting the need to address specific caries prevention strategies at each dentition phase in immunocompromised children.

While a wide range of caries prevalence has been reported in school-aged children in sub-Saharan Africa, several studies report estimates ranging 10–22% [11, 31–34]. Results from several studies of children perinatally infected with HIV, including ours of early childhood [4, 20], are consistent with our findings of a higher caries prevalence in HIV-infected children [2, 3, 8, 13, 17, 18, 35–41]. While a non-comparative study [42] observed a low prevalence of early childhood caries in children with perinatal HIV infection, several comparative studies [4, 13, 25, 37, 43] have reported elevated risk of dental-related diseases, including caries, in PLWH compared to their uninfected counterparts. The mechanism behind this elevated caries risk associated with HIV infection is not clear and has not been adequately investigated. Studies in younger children suggest that HI children are more susceptible to ECC when compared to their HIV-uninfected counterparts due to an increased level of *Streptococcus mutans* [44, 45]. *Candida albicans* also has been implicated in driving the risk of dental caries in PLWH by facilitating the colonization of *S. mutans* [46]. Significant changes in salivary microbial composition have been reported between in PLWH when compared with people not living with HIV, likely increasing the risk for caries in PLWH [20, 47]. The persistently defective immune responses or chronic immune activation experienced in PLWH on ART could also play a significant role in increasing this risk. There is correlation between low levels of CD4 + T cells and caries severity [3], gingivitis [48] and xerostomia and hyposalivation [49, 50]. People living with HIV are susceptible to xerostomia and hyposalivation due to adversely affect salivary gland output [51, 52]. While the etiology of hyposalivation and xerostomia associated with caries has been well-described [45], we were only able to assess hyposalivation as many children did not report a symptom of dry mouth/xerostomia. In addition, xerostomia can occur with hyposalivation [54, 55] or without hyposalivation [45] and be associated with caries. Also, some syrup- or suspension-based antiretroviral medications are sucrose-based [1, 53]. While most HI children in our study were receiving zidovudine-based regimens (AZT/3TC/NVP or EFV) as their first-line regimens, integrase-inhibitor dolutegravir-based regimens are currently being rolled out for children in Nigeria [54]. This is particularly important as the US Pediatric HIV/AIDS Cohort Study (PHACS) cohort had previously reported a higher caries risk among youth receiving integrase-inhibitors, compared to those who did not [38]. Furthermore, PHACS cohort reported lower dmft scores in children who initiated protease inhibitors (PIs) before age 6 when compared to those who did not initiate PIs before age 6 [38]. Such data adds to growing evidence that PI-based ART confers favorable clinical and immunological outcomes [55].

All these aforementioned factors, in addition to other dietary and socioeconomic related factors, could explain the higher prevalence of caries observed in HI children that may differentiate the study groups [56, 57]. These factors require further examination in future studies.

Perinatal HIV exposure alone did not appear to increase the risk of dental caries in this population as observed in other studies [1, 9]. In fact, in the younger age group (≤ 6 years), we observed a lower prevalence of caries in the primary teeth of HEU children compared to their unexposed counterparts. While this observation needs to be further investigated based on our previous report [4], there are several reasons that could explain this finding. It is likely that HUU children in this study population might have heightened levels of unmeasured risk factors that include nutritional status, immunological markers and genetics associated with caries compared to HI and HEU children. In addition, although speculative, it is reasonable to posit as a hypothesis that the development and training of the immune system via the oral microbiota [20], and perhaps the gut microbiota [58], in HEU children via maternal ART exposure in-utero and lower likelihood of breast feeding (observed in mothers of HEU children) might be protective of caries [4]. Our thoughts are based on results from a previous analysis of younger children suggesting a higher odds of caries in children delivered after a spontaneous membrane rupture and in those who were breastfed for longer durations [4]. It is very interesting to note that in that study, HEU children were least likely to be delivered via spontaneous membrane rupture or vaginal delivery. These findings however require further investigations.

Given our previous work that showed immune status (CD4 lymphocyte counts) had a stronger impact (compared to HIV infection) on caries [4] and the salivary microbiota [20], we examined the association between CD4 counts or percentages and caries experience without including HIV infection and exposure status in the models. In these models, low CD4 + cell counts were consistently associated with higher caries prevalence and severity. While salivary pH levels were not assessed in this study, our observations suggest that a higher salivary pH level, is essential for the prevention of dental caries, and is associated with a better immune status, while a worse dental health status is closely associated with a poorer immune status. A prior study has indicated this possibility [59]. Notably, impaired immunity or immunodeficiency may lead to a decrease in salivary flow, impeding the recovery of the salivary pH level after eating [59]. This phenomenon has been reported in patients with other immunodeficiency associated diseases such as end-stage renal disease [60].

A key strength of this study is the utilization of a large sample of age- and sex-matched HI, HEU and HUU children living in sub-Saharan Africa. The large sample size addressed the question of whether HIV infection increases the risk of caries, allowed for stratified analyses and provides novel insights into age- and dentition-specific patterns of association. Uniform CD4 assessments were done across all 3 study groups, irrespective of HIV exposure or infection status—this is another unique feature of the present investigation, as many studies do not measure CD4 levels in uninfected subjects. Still, this study has several limitations. The main limitation is that we were unable to separate the effect of ART from that of HIV infection on caries experience, because all HI children were on ART (i.e., era of *treat all*) and received the similar regimens due to availability/drug supply chain. Moreover, we did not record morphological extent of the caries, whether caries lesions were occlusal or interproximal. Also, without radiographic assessments, we were not able to determine lesions with pulp involvement thereby limiting the full range of evaluation of the caries severity. However, our examiners were well-calibrated to ensure reliability of the caries assessments and arguably, only early-stage proximal lesions might be systematically missed without radiographs, as other lesions would be clinically visible. While dietary information was not included in this analysis, it would be foundational for future studies to include a validated dietary scale in sub-Saharan Africa. Finally, while data from this present cohort study reflect only baseline assessments, our cross-sectional approach provides a foundation for subsequent longitudinal assessments.

## Conclusion

This study’s findings suggest that HIV status is associated with higher prevalence and severity of dental caries mainly in the permanent dentition; and the impact of HIV infection and exposure may differ with age/dentition phase and HIV exposure. Building upon earlier reports, here we provide additional evidence, using a sizeable community-based cohort of HI, HEU, and HUU children. Our findings suggest the need for specific and targeted oral health care for children who are HI, HEU and HUU with respect to caries prevention in early and middle childhood. Finally, this study lays the foundation for investigating and identifying key contributory factors, including environmental, microbial and dietary factors, that may contribute to caries development in children exposed to and infected with HIV.

## Supplementary Information

**Additional file 1: Table S1**. Summary of Oral and Dental Characteristics by Study Group. **Table S2**. Odds of dental caries by CD4 levels: Results from unadjusted and adjusted logistic regression models. **Table S3**. Association between caries burden and CD4 counts: Results from negative binomial models.

## Data Availability

Questionnaires, clinical data and associated dataset generated and/or analyzed for this current study cannot be made publicly available as required consent to publish data was not given. However, deidentified data can be made available from the corresponding author on reasonable request.

## Abbreviations

ANOVA: Analysis of variance
ART: Antiretroviral therapy
CD4 cells: Glycoproteins associated with T cell receptors especially on the surface of helper T cells
DOMHaIN: Dental caries and its association with oral microbiomes and HIV in young children—Nigeria
HIV: Human immunodeficiency virus
HI Children: Children with perinatally acquired HIV infection
HEU Children: Children perinatally exposed to HIV
HUU Children: Children with were neither perinatally exposed or infected with HIV
ICDAS: International caries detection and assessment
PLWH: People living with HIV
