# Supplementary material for: Age-specific associations with dental caries in HIV-infected, exposed but uninfected and HIV-unexposed uninfected children in Nigeria
Source: BMC Oral Health. 2022 Sep 27;22:429. doi: 10.1186/s12903-022-02421-w (PMC9512979; doi:10.1186/s12903-022-02421-w)
Supplement: Supplementary file 1 — Additional file 1: Table S1. Summary of Oral and Dental Characteristics by Study Group. Table S2. Odds of dental caries by CD4 levels: Results from unadjusted and adjusted logistic regression models. Table S3. Association between caries burden and CD4 counts: Results from negative binomial models. [file 12903_2022_2421_MOESM1_ESM.docx]

**Age-Specific Associations with Dental Caries in HIV-Infected, Exposed but Uninfected and HIV-Unexposed Uninfected Children in Nigeria**

# Supplementary Table 1. Summary of Oral and Dental Characteristics by Study Group

**Supplementary Table 2.** Odds of dental caries by CD4 levels: Results from unadjusted and adjusted logistic regression models

# Supplementary Table 3. Association between caries burden and CD4 counts: Results from negative binomial models

# Supplementary Table 1. Summary of Oral and Dental Characteristics by Study Group

|  | **All (N=544)** | **HI (N=181)** | **HEU (N=177)** | **HUU (N=186)** | **Overall^Ω^**  **P value** |
| --- | --- | --- | --- | --- | --- |
| **Caries prevalence, n(%)** | 129 (24) | 60 (33) | 26 (15) | 43 (22) | **<0.0001** |
| dmft > 0, n(%) | 115 (21) | 52 (29) | 22 (12) | 41 (22) | **<0.0001** |
| Mean dmft, mean (sd) | 2.63 (1.94) | 2.81 (2.0) | 2.32 (1.96) | 2.56 (1.8) | 0.59 |
| DMFT > 0, n(%) | 35 (6) | 21 (12) | 8 (4) | 6 (3) | **0.002** |
| Mean DMFT, mean (sd) | 2.34 (1.6) | 2.86 (1.8) | 1.87 (1.1) | 1.17 (0.4) | **0.05** |
| No of maintained primary teeth, mean (sd) | 8.35 (6.6) | 8.07 (6.6) | 8.39 (6.4) | 8.59 (6.9) | 0.75 |
| No of erupted permanent teeth, mean (sd) | 13.95 (5.3) | 14.27 (5.2) | 13.9 (5.1) | 13.69 (5.4) | 0.58 |
| Enamel defects, n(%) | 48 (22) | 22 (12) | 13 (8) | 13 (7) | 0.17 |
| Oral Hygiene Index, mean (sd) | 1.04 (0.7) | 1.08 (0.7) | 1.07 (0.8) | 0.96 (0.6 ) | 0.21 |
| Gingival Index, mean (sd) | 0.22 (0.4) | 0.24 (0.4) | 0.22 (0.4) | 0.21 (0.3) | 0.76 |
| Hyposalivation, n(%) | 11 (20.2) | 9 (5) | 1 (0.6) | 1 (0.5) | **0.003** |

^Ω^ Group ANOVA, dmft/DMFT- Decayed, missing, filled teeth, sd- standard deviation

# Supplementary Table 2. Odds of dental caries by CD4 levels: Results from unadjusted and adjusted logistic regression models

|  | **Caries in Any Dentition** | **Caries in Primary Dentition** | **Caries in Permanent Dentition** |
| --- | --- | --- | --- |
|  | **Adjusted OR**  **(95% CI)** | **Adjusted OR (95% CI)** | **Adjusted OR (95% CI)** |
| **CD4 count** |  |  |  |
| <=500 cells/mm^3^ | **1.95 (1.19 -3.19)** | **2.16 (1.31 -3.57)** | **2.04 (0.90 - 4.64)** |
| >500 cells/mm^3^ | ref | ref | ref |
| Sex |  |  |  |
| Female | 0.90 (0.59-1.36) | 0.86 (0.56–1.33) | 0.66 (0.30 –1.42) |
| Male | ref | ref | ref |
| Age (in months) | **1.01 (1.00-1.02)** | 1.00 (0.99-1.01) | **1.04 (1.02-1.06)** |
| Gingival Inflam. Score | **1.89 (1.22-2.91)** | **1.75 (1.12-2.74)** | 1.88 (1.84-4.21) |
| Hypoplastic teeth | **3.21 (1.70- 6.06)** | **3.21 (1.69- 6.11)** | **3.63 (1.40 - 9.37)** |
| Low salivary flow rate | 0.80 (0.19-3.31) | 0.56 (0.12-2.61) | 2.84 (0.50-16.03) |
| No Prior Visit to a Dentist | **3.36 (1.07- 10.57)** | **2.87 (0.88-9.41)** | 3.22 (0.55-18.90) |

*p values <0.1 are in bold fonts*

# Supplementary Table 3. Association between caries burden and CD4 counts: Results from negative binomial models

|  | **# of carious teeth in**  **Any Dentition** | **# of carious teeth in**  **Primary Dentition** | **# of carious teeth in**  **Permanent Dentition** |
| --- | --- | --- | --- |
|  | **Adjusted IRR**  **(95% CI)** | **Adjusted IRR**  **(95% CI)** | **Adjusted IRR**  **(95% CI)** |
| CD4 count |  |  |  |
| <=500 cells/mm^3^ | **1.75 (1.01-3.02)** | 1.55 (0.86-2.77) | **3.27 (1.17-9.16)** |
| >500 cells/mm^3^ | ref | ref | ref |
| Sex |  |  |  |
| Female | 0.96 (0.62-1.47) | 0.92 (0.58-1.46) | 0.92 (0.38-2.25) |
| Male | ref | ref | ref |
| Age (in months) | 1.00 (0.99-1.01) | 1.00 (0.99-1.01) | **1.06 (1.03-1.09)** |
| Gingival Inflammation |  |  |  |
| Yes | **1.74 (1.10 – 2.73)** | **1.73 (1.08 – 2.79)** | 1.75 (0.68 – 4.51) |
| No |  |  |  |
| Enamel defects (hypoplasia) | **2.54 (1.22 - 5.28)** | **2.77 (1.24 – 6.18)** | **4.00 (1.02-15.67)** |
| Low salivary flow rate | 1.02 (0.22-4.61) | 0.57 (0.11 – 2.96) | 2.97 (0.19-47.52) |
| No Prior Visit to a Dentist | 3.09 (0.79-12.19) | 2.02 (0.48 – 8.49) | 2.23 (0.68 – 4.51) |

IRR = incidence risk ratio (estimating prevalence ratios); CI = confidence interval.

p values <0.1 are in bold fonts
